# Extended use or reuse of single-use surgical masks and filtering face-piece respirators during the coronavirus disease 2019 (COVID-19) pandemic: A rapid systematic review

**DOI:** 10.1017/ice.2020.1243

**Published:** 2020-10-08

**Authors:** Elaine C. Toomey, Yvonne Conway, Chris Burton, Simon Smith, Michael Smalle, Xin-Hui S. Chan, Anil Adisesh, Sarah Tanveer, Lawrence Ross, Iain Thomson, Declan Devane, Trish Greenhalgh

**Affiliations:** 1 School of Allied Health, University of Limerick, Limerick, Ireland; 2 Evidence Synthesis Ireland, School of Nursing and Midwifery, National University of Ireland, Galway, Ireland; 3 Academic Unit of Primary Medical Care, University of Sheffield, Sheffield, United Kingdom; 4 Canadian Standards Biological Aerosols Working Group, Canada; 5 James Hardiman Library, National University of Ireland, Galway, Ireland; 6 Centre for Tropical Medicine and Global Health, Nuffield Department of Medicine, University of Oxford, Oxford, United Kingdom; 7 Division of Occupational Medicine, Department of Medicine, University of Toronto and St Michael’s Hospital, Unity Health, Toronto, Canada; 8 Department of Pharmaceutical Health Services Research, University of Maryland, Baltimore, Maryland, United States; 9 Department of Infectious Disease, Children’s Hospital of Los Angeles, Los Angeles, California, United States; 10 Médecins Sans Frontières/Doctors without Borders, Geneva, Switzerland; 11 Nuffield Department of Primary Care Health Sciences, University of Oxford, Oxford, United Kingdom

## Abstract

**Background:**

Shortages of personal protective equipment during the coronavirus disease 2019 (COVID-19) pandemic have led to the extended use or reuse of single-use respirators and surgical masks by frontline healthcare workers. The evidence base underpinning such practices warrants examination.

**Objectives:**

To synthesize current guidance and systematic review evidence on extended use, reuse, or reprocessing of single-use surgical masks or filtering face-piece respirators.

**Data sources:**

We used the World Health Organization, the European Centre for Disease Prevention and Control, the US Centers for Disease Control and Prevention, and Public Health England websites to identify guidance. We used Medline, PubMed, Epistemonikos, Cochrane Database, and preprint servers for systematic reviews.

**Methods:**

Two reviewers conducted screening and data extraction. The quality of included systematic reviews was appraised using AMSTAR-2. Findings were narratively synthesized.

**Results:**

In total, 6 guidance documents were identified. Levels of detail and consistency across documents varied. They included 4 high-quality systematic reviews: 3 focused on reprocessing (decontamination) of N95 respirators and 1 focused on reprocessing of surgical masks. Vaporized hydrogen peroxide and ultraviolet germicidal irradiation were highlighted as the most promising reprocessing methods, but evidence on the relative efficacy and safety of different methods was limited. We found no well-established methods for reprocessing respirators at scale.

**Conclusions:**

Evidence on the impact of extended use and reuse of surgical masks and respirators is limited, and gaps and inconsistencies exist in current guidance. Where extended use or reuse is being practiced, healthcare organizations should ensure that policies and systems are in place to ensure these practices are carried out safely and in line with available guidance.

The COVID-19 pandemic has put global healthcare systems under unprecedented strain and has exposed healthcare workers in a wide range of clinical environments to risk of infection.^1,2^ Infection prevention and control measures developed for healthcare workers recommend personal protective equipment (PPE), including surgical masks and respirators, as part of a broader hierarchy of protective measures.^3^

Global shortages have forced the consideration of PPE-sparing measures, including extended use, reuse, and reprocessing of single-use masks and respirators.^4,5^ Extended use is the practice of using the same single-use mask or respirator for encounters with multiple patients without removing it.^6^ Reuse is using the same mask or respirator for multiple encounters with patients, removing it (‘doffing’) for storage after each encounter, and putting it on again (‘donning’) prior to the next encounter with a patient.^6^ Reprocessing is ‘decontamination using disinfection or sterilization methods followed by reuse of either reusable or disposable PPE.’^7^ When applied to single-use masks and respirators, each practice can potentially lead to reduced respiratory protection, comfort, and safety for healthcare workers (Fig. 1). Recent research found that healthcare workers reporting reuse of PPE had a 46% increased risk of reporting a positive severe acute respiratory coronavirus virus 2 (SARS-CoV-2) test compared to those with adequate equipment.^8^

**Fig. 1.**
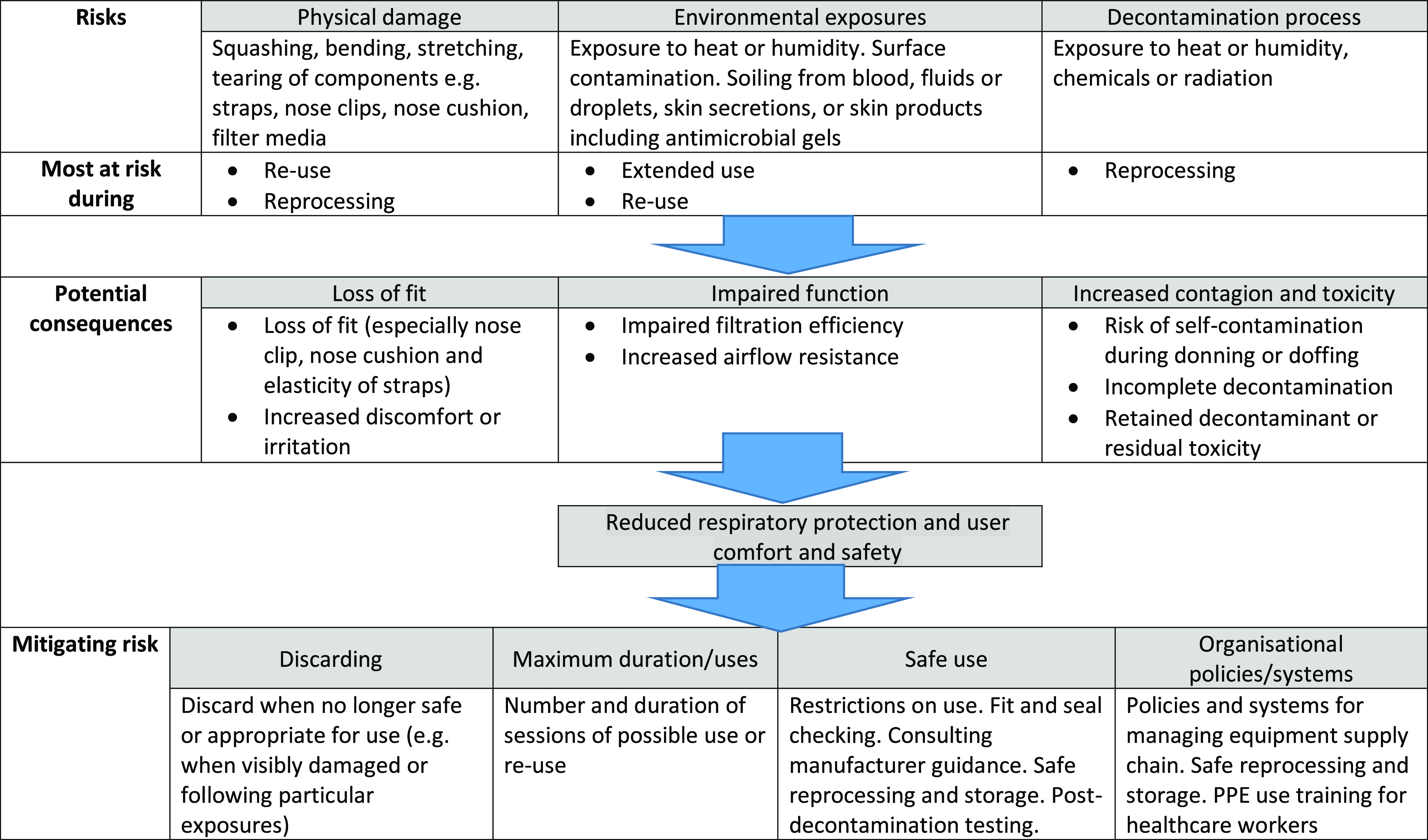
Taxonomy of potential risks and mitigation with respect to extended use/re-use/reprocessing of single-use masks and respirators

Several national and international guidelines make reference to extended use, reuse, and reprocessing of single-use masks and respirators.^9^ We compared these guidelines first with each other and second with current synthesized evidence, particularly in the light of current worldwide shortages of PPE to inform rapidly evolving policies and practice.

## Methods

We conducted a rapid review in line with Cochrane Interim Guidance for Rapid Reviews,^10^ reported according to PRISMA reporting criteria (Supplementary File 1 online).^11^ The protocol^12^ was published on May 5, 2020, before completion of data extraction.

## Literature searches

### Identification of guidance

We conducted a targeted search of major international and national health organization websites including the World Health Organization (WHO), the European Centre for Disease Prevention and Control (ECDC), the US Centers for Disease Control and Prevention (CDC), and Public Health England (PHE) between March 23 and May 22, 2020, to identify current guidance on reuse or extended use of surgical masks or filtering face-piece respirators. The identified documents were screened for inclusion and verified.

### Identification of systematic reviews

We sought to identify systematic reviews of primary studies exploring any aspect of the extended use, reuse, or reprocessing of any type of surgical mask or filtering face-piece respirator on outcomes including technical performance standards, decontamination outcomes, or impact on healthcare workers (eg, health outcomes or qualitative outcomes such as acceptability). Systematic electronic database searches of Medline, the Cochrane Database of Systematic Reviews, Epistemonikos, and PubMed were conducted by an experienced information specialist on April 28, 2020. This procedure was supplemented with searches of preprint repositories Litcovid, medRxiv, and Open Science Framework. We scanned reference lists of included documents and contacted authors of included reviews to identify additional records. No date limit or language restrictions were applied. Search terms included (masks OR respiratory devices) AND (infection control OR decontamination) AND (reuse OR extended use) (see Supplementary File 2 online for search strategy). A systematic review search filter was not applied in Medline but was applied in Epistemonikos, the Cochrane Database, and PubMed to minimize duplication and increase specificity. Records were imported into Covidence and were double-screened by 2 reviewers independently at the title and abstract stage and at the full-text stage.

### Data extraction and synthesis

Data were extracted and verified using predeveloped data extraction templates in Excel (Microsoft, Redmond, WA) and NVIVO (QSR International, Doncaster, Australia). Data extraction fields included categories (eg, organization or country) and free text (eg, definitions of terms, details of recommendations, and review findings). Data extracted from guidelines were tabulated according to recommendations regarding extended use, reuse, and reprocessing. No quality appraisal was undertaken on guidelines because the purpose of this part of the review was to document recommendations. Included systematic reviews were assessed independently for quality by experienced systematic reviewers using AMSTAR 2. Data extracted from systematic reviews were tabulated to allow comparisons across reviews and with guidance documents.

Extracted data were analyzed using a narrative synthesis approach with a critical to ensure that the narrative synthesis reflected original findings of source documents. Critical input was also provided by experts in infection control, occupational medicine, and PPE to ensure relevance and applicability.

## Results

**Fig. 2.**
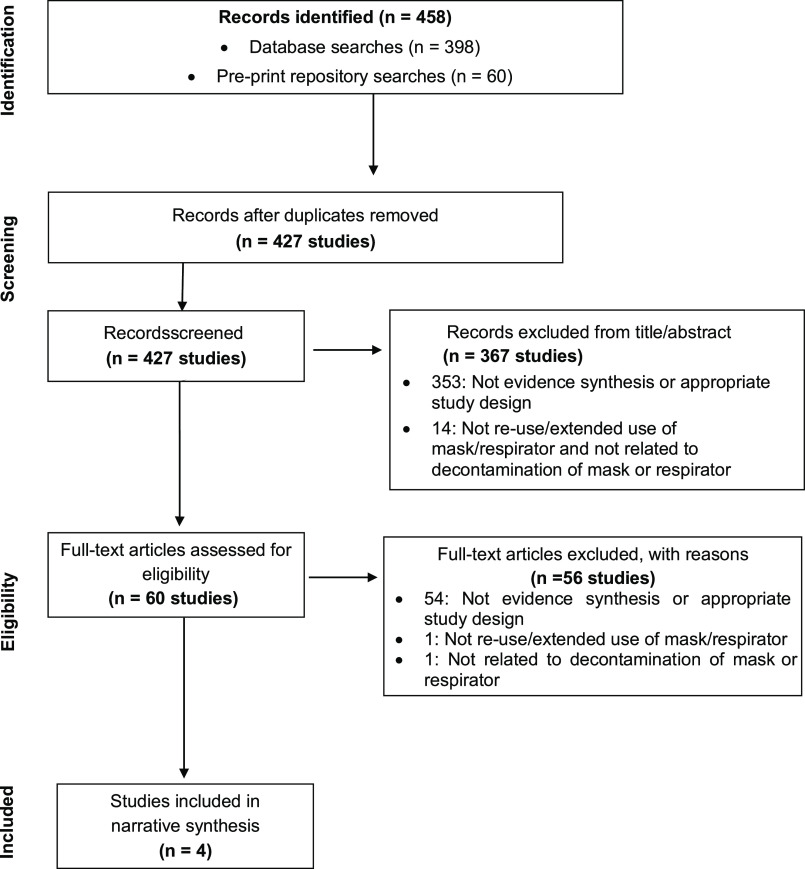
PRISMA flow diagram

Overall, 6 documents were identified that provided guidance on the extended use, reuse, and/or reprocessing of surgical masks or filtering face-piece respirators (overview provided in Supplementary File 3 online). The search for reviews retrieved 458 records, and 60 full-text articles were screened; 4 relevant systematic reviews were included (Fig. 2).

### Summary of guidance

We reviewed 3 guidance documents from the US CDC^6,13,14^ and 1 each from the ECDC,^15^ the PHE,^16^ and the WHO.^7^ All were published or updated between March 17, 2020, and May 21, 2020, and were written for the COVID-19 pandemic. None included a systematic literature search, and the depth of referencing and level of detail varied. All guidance documents depicted extended use, reuse, or reprocessing of single-use masks and respirators as extraordinary, last-resort measures to be considered only during a critical shortage of equipment when other strategies for rational use of supplies have been exhausted (eg, minimizing need for PPE through administrative and engineering controls and coordinating supply-chain management). The US CDC, the WHO, and PHE recommend additional procedures at the organizational level: appropriate documentation and recording of reuse or reprocessing, quality assurance of reprocessing measures, suitable reprocessing and storage facilities and systems, and staff training regarding safe use and donning or doffing of masks or respirators if reusing or extending use.

All guidance documents favored extended use over reuse because of reduced risk of contact transmission. All recommended ensuring good fit, performing a seal check, and inspecting for function and potential damage prior to use or reuse of any mask or respirator. The US CDC and the WHO acknowledged that face masks and respirators that have passed their expiry date may sometimes be used in situations of limited capacity. Table 1 summarizes guidance on the extended use and reuse of surgical masks; findings are grouped by the 3 forms of risk mitigation shown in Figure 1. Table 2 summarizes guidance on extended use and reuse of respirators. Table 2 also summarizes guidance on reprocessing of respirators, which were framed with caution to reflect the high degree of uncertainty and potential risk.

**Table 1.** Summary of Guidance Recommendations for Fluid-Resistant Surgical Masks

Guidance	CDC	ECDC	PHE^a^	WHO
**Extended use**				
Discarding	Soiled, damaged or hard to breathe through	No information	Moist, damaged, visibly soiled or difficult to breathe through	Wet, soiled, splashed, damaged or difficult to breathe through If displaced from face or touched
Maximum duration	Not specified		2–6 h dependent on setting and activity	Up to 6 h
Safe use	Hand hygiene if adjusted or touched Only continue within cohorted area		Hand hygiene if adjusted or touched Discard after leaving cohorted area unless transferring a patient.	Only continue within cohorted area. Discard on leaving.
**Reuse**				
Discarding	Soiled, damaged or hard to breathe through.	Not recommended	Moist, damaged, visibly soiled or difficult to breathe through	No information
Maximum uses	Not specified		Not specified	
Safe use	Do not reuse masks that fasten with ties (elastic ear hooks more suitable). Fold^1^ carefully and store in sealable paper bag or breathable container between uses.		Masks with elastic ear hooks more suitable for reuse Fold^b^ carefully and store in labelled sealable bag between uses. Some models cannot be reused as they deform once being donned.	
**Reprocessing**				
DiscardingMaximum duration/usesSafe use	Not recommended			

Note. CDC, Centers for Disease Control and Prevention; ECDC, European Centers for Disease Control and Prevention; PHE, Public Health England; WHO, World Health Organization.

a PHE guidelines on extended use and reuse do not clearly differentiate between surgical masks and filtering face-piece respirators.

b “Fold” cannot apply to molded-cup–type face masks.

**Table 2.** Summary of Guidance Recommendations for Filtering Face-Piece Respirators

Guidance	CDC	ECDC	PHE^a^	WHO
**Extended use**				
Discarding	Soiled (blood, secretions, body fluids), if obvious damage or difficult to breathe through Discard after use in aerosol-generating procedure.	Soiled, wet, can no longer be properly fitted or difficult to breathe through Discard after use in aerosol-generating procedure	Moist, damaged, visibly soiled or difficult to breathe through	Wet, soiled, splashed, damaged or difficult to breathe through If displaced from face or touched
Maximum duration	Not specified (notes some studies up to 8 h), guided by hygienic/practical concerns	Time guided by hygienic/practical concerns^b^	Not specified^c^	Up to 6 h
Safe use	Hand hygiene if adjusted or touched Respirator can be covered with face shield (‘strongly preferred’) or surgical mask. Discard after leaving the cohorted area.	Respirator can be covered with a face shield or medical mask to extend use.	Hand hygiene if adjusted or touched Discard after leaving cohorted area unless transferring a patient.	Only continue within cohorted area. Discard after leaving.
**Reuse^d^**				
Discarding	Soiled (blood, secretions, body fluids) If obvious damage or difficult to breathe through Discard after use in aerosol-generating procedure.	No information	Moist, damaged, visibly soiled or difficult to breathe through	Reuse without reprocessing strongly discouraged
Maximum uses	Up to 5 times unless manufacturer states otherwise		Not specified	
Safe use	(a) Ensure safe storage and limit to named user (b) Follow 1 day of use with 4 day “quarantine” period in labelled breathable sealed container before reuse^e^		Fold and store in labelled sealable bag between uses. Some models cannot be reused because they deform once donned.	
**Reprocessing^d^**				
Discarding	If integrity of any component (eg, straps, bridge) of the respirator is compromised, or if a successful user seal check cannot be performed.	Not specified	Currently no recommendations (but acknowledge work on validating methods in progress)	Check integrity and shape before reprocessing. Discard if damaged/not suitable for reuse.
Maximum duration or uses	Not specified	Not specified		“After a predefined number of uses”
Safe use	Not for use in aerosol-generating procedures^f^	Quality checks of reprocessing necessary to ensure safety		After use, label and place in reprocessing container. Reprocessing performed by trained staff Return to original wearer after reprocessing.

Note. CDC, US Centers for Disease Control and Prevention; ECDC, European Centre for Disease Prevention and Control; PHE, Public Health England; WHO, World Health Organization.

a PHE guidelines on extended use and reuse do not clearly differentiate between surgical masks and filtering face-piece respirators

b “… can be re-used for a limited time, unless there is a risk for contamination through the deposition of infectious particles on the surface.”

c States that filter capacity of FFP3/FFP2/N95 respirators greatly exceeds one day of use in healthcare or social care.

d Reused and reprocessed respirators are likely to be used for extended periods, in this case the extended use criteria also apply.

e Currently appears in decontamination guidelines as an alternative to decontamination but does not appear in the reuse guideline.

f Unless manufacturer information indicates that decontamination does not affect performance.

### Systematic reviews

The 3 systematic reviews were conducted between March and April 2020 and at the time of submission were preprints or were under peer review. All were conducted in Canada; 3 were conducted by the same research team. These 3 focused on reprocessing of filtering face-piece respirators using different decontamination interventions (ie, heat-based treatments, disinfectant, and ultraviolet germicidal irradiation),^17–19^ and 1 study covered reprocessing of surgical masks and ‘precontamination’ interventions applied before use to enable extended use or reuse. No reviews in our sample examined the impact of extended use or reuse of filtering face-piece respirators or surgical masks on the ability to meet technical standards or on healthcare worker acceptability outcomes such as comfort. The AMSTAR 2 ratings for each study are provided in Supplementary File 4 (online). The included reviews were judged to be predominantly of high quality. However, none provided a list of excluded studies or justified the reasons for exclusion, and no study reported on the sources of funding for included primary studies.

Detailed descriptions of the reviews and their findings are provided in Supplementary File 5 (online). The 3 reviews on filtering face-piece respirators included between 11 and 13 studies (28 unique studies in total). The included studies evaluated the effects of reprocessing on various outcomes including effective decontamination, appearance, performance (including filtration efficiency and airflow resistance), user comfort, fit, and safety (ie, increased contagion risk to healthcare workers due to insufficient decontamination and/or toxicity due to residual decontaminant). In relation to decontamination, most of the evidence in the reviews supported vaporized hydrogen peroxide, moist/dry heat (range, 60–90°C), and ultraviolet germicidal irradiation interventions. The studies included in the reviews were generally at low risk of bias. However, few primary studies investigated the impact of these methods on fit or user comfort, and there was substantial variability in the models of filtering face-piece respirator used across studies. Only 2 studies^20,21^ included in 1 review^19^ explored the effect of reprocessing on SARS-CoV-2. In addition, 7 studies were included in the review of surgical mask decontamination, but only 1 of these had specifically evaluated decontamination interventions after use to enable reuse. The review concluded that there was insufficient evidence regarding the safety or efficacy of any decontamination intervention for reprocessing surgical masks.

### Comparison of guidance and systematic reviews on reprocessing of masks or respirators

Table 3 compares the findings of the systematic reviews with the 3 guidance documents relating to the reprocessing of surgical masks and respirators. There is considerable discrepancy to the extent that no single reprocessing method is supported by all the guidance documents. The intervention with most support is vaporized hydrogen peroxide, though one document cautions about chemical residues and another indicates that it has only been tested with some of the respirator models in common use. Similarly, ultraviolet germicidal irradiation receives both cautious support and concerns about inadequate decontamination because of incomplete penetration into deeper layers of the filter. Moist heat is cited as promising, though there are concerns when steam is microwave generated because it may be uneven heating and the metal nose-band may generate sparks.

**Table 3.** Summary of Systematic Review Conclusions Compared to Guidance Recommendations for Reprocessing Methods^a^

Type	Method	Review	Review conclusion	CDC^b^	ECDC	WHO
**Filtering face-piece respirators**						
Microwave & heat based treatments	Microwave (dry/moist), 60–90°C	Gertsman 2020	Effective sterilization while maintaining mask integrity (some models)	Moist heat is promising Steam treatment is promising with some limitations: variability of microwaves used to generate steam, sparking concerns Dry microwave irradiation/dry heat not recommended	Not mentioned	Not recommended Inconsistency of machines Overheating/sparking of metal nose band
	Other heat (dry/moist), 60–90°C		Effective sterilization while maintaining mask integrity (some models)		Steam sterilization at temperatures <134°C under study	Steam sterilization at 134°C not recommended
	Autoclaving/heat >90°C		Not recommended Damage to mask integrity	Not recommended	Not recommended	
Chemical disinfectants	Vaporized hydrogen peroxide	O’Hearn 2020a	Removes pathogens without affecting function or fit Minimal impact on appearance	Promising^b^	Cautiously cites supportive studies Concern about residual chemicals and filtration	Cautious support Limited number of models tested
	Liquid hydrogen peroxide		Further research needed on decontamination effects and impact on fit	Promising with some limitations	Not mentioned	Not mentioned
	Sodium hypochlorite		Not recommended Adverse effect on function	Not recommended	Not mentioned	Not recommended
	Ethylene oxide		Not recommended Potential health risk	Not recommended Potentially harmful	Mentioned but no recommendation	Cautious support Limited number of models tested
	Ethanol/isopropyl alcohol		Not recommended	Not recommended	Not mentioned	Not recommended
Other methods	UV germicidal irradiation	O’Hearn 2020b	Effective decontamination of mask surfaces while maintaining mask integrity (some models)	Promising^b^ Efficacy dose dependent upon proper precautions are required to avoid UVGI exposure to skin or eyes.	Mentioned but no recommendation	Cautious support Concerns about penetration of UV light to deeper layers of filter
	Disinfectant wipes	…	No systematic review evidence	Not recommended	Not mentioned	Not mentioned
	Gamma irradiation	…	No systematic review evidence	Not mentioned	Cautiously cites ongoing studies Concerns about availability and impact on fit	No evidence in masks/respirators
	Ozone decontamination	…	No systematic review evidence	Not mentioned	Mentioned but no recommendation	Not mentioned
**Surgical masks**						
All methods	Dry and moist heat, ethanol, isopropanol, sodium hypochlorite (single studies only)	Zorko 2020	Inadequate evidence on the safety or efficacy of any method	Not recommended	Not recommended	Not recommended

Note. CDC, US Centers for Disease Control and Prevention; ECDC, European Centre for Disease Prevention and Control; PHE, Public Health England; WHO, World Health Organization; UV, ultraviolet.

a No data are given for PHE because no recommendations have been made in their guidance.

b The CDC identified these methods as showing most promise and recommends focusing current efforts on these technologies.

### Inconsistencies and gaps in evidence

Our review of guidance documents and systematic reviews highlights several inconsistencies that warrant exploration, even though the COVID-19 pandemic presents unique challenges for different contexts. This is particularly true of the emerging science of reprocessing respirators, for which authorities appear to have proceeded with different degrees of caution in making recommendations.

Although the ECDC recommends the addition of either a surgical mask or a cleanable face shield over a respirator, the US CDC strongly recommends a face shield rather than a surgical mask due to supply issues and concerns that a surgical mask could affect the function of the N95 respirator. Moreover, evidence suggests that the addition of a surgical mask does not improve respiratory protection and may increase user discomfort and impair communication.^22,23^

The US CDC and ECDC make specific recommendations regarding respirators and aerosol-generating procedures. Both recommend discarding and not reprocessing respirators after use in aerosol-generating procedures, and the US CDC also recommends not using a reprocessed respirator while carrying out an aerosol-generating procedure (subject to manufacturer guidance). However, the WHO and PHE do not explicitly recommend that respirators be discarded after aerosol-generating procedures, nor do they mention the use of reprocessed respirators in aerosol-generating procedures. No guidance mentions the use or discarding of respirators in areas where aerosols may be present.

Guidance regarding the reuse of respirators without reprocessing is inconsistent and lacks clarity. The WHO guidance strongly discourages the reuse of respirators without appropriate decontamination. However, citing a recent study by Van Doremalen et al^24^ suggesting that SARS-CoV-2 viral particles may remain infective for up to 72 hours, the US CDC suggests a system in which each healthcare worker is allocated 5 respirators for daily use in strict rotation, using careful storage to essentially ‘quarantine’ the devices. The US CDC suggest that if 5 respirators are not available for each worker, then decontamination may be necessary, potentially suggesting that this should be an initial strategy prior to attempting reprocessing. We found no systematic review evidence testing this approach, and neither PHE or ECDC explicitly discuss reuse with or without reprocessing. Moreover, data from the study cited showed that the 72-hour period was just the length of the measurement period and that survival followed a decay curve, not a fixed “all dead” time, implying that the length of time an amount greater than or equal to the infective dose survives depends on the starting quantity of virions.^24^

The systematic reviews included laboratory studies to test whether reprocessing changed the properties of a respirator. None of the reviews or research reported in guidance documents described practical or operational studies of facilitating reuse or reprocessing of respirators at scale. This is a key gap in the research, given the existing strain on healthcare resources and the requirement to ensure that a respirator remains matched to a healthcare worker throughout its use.

## Discussion

Our systematic review yielded 5 key findings. First, while extended use or reuse of single-use surgical masks or respirators (with or without reprocessing) is generally not recommended, guidance from various organizations supports such measures (preferably extended use rather than reuse) as a last-resort measure during critical shortage. Second, comparisons across guidance documents and systematic reviews highlight limited evidence, varying levels of detail, and areas of inconsistency, especially in relation to the reuse of respirators (with or without reprocessing) during and after aerosol-generating procedures. Third, the reprocessing of surgical masks is not recommended. Fourth, the reprocessing of respirators under controlled and standardized conditions is recommended, but there is inconsistency regarding how or when this should take place. Fifth, where extended use or reuse is being practiced, healthcare facilities and institutions should ensure that policies and systems are in place to enable these practices to be carried out in the safest way possible in line with available guidance.

### Meaning of the study: implications for clinicians and policy makers

Our findings highlight the numerous risks related to the extended use, reuse, or reprocessing of single-use surgical masks and respirators. The guidance is unanimous that these practices should be considered only in situations of extreme critical shortage, after all other strategies have been employed to minimize strain on supply. Where extended use or reuse is unavoidable, risks should be carefully assessed and policies and decision making should be made on the best available evidence. Where evidence is lacking or unclear, difficult judgments need to be made to balance current safety of staff regarding the conservation of supply and the future protection of staff. Given the rapidly growing body of related research, guidance should be regularly reviewed and updated.

Surgical masks and respirators have different properties and functions. They should be distinguished clearly in policies and guidance, which should also take into consideration the variability of respirator models and manufacturer guidance. Policies should also be developed that address reuse clearly in relation to different reprocessing methods, and also address use and reuse of respirators in different situations, for example, during and after aerosol-generating procedures. Policies on reuse and extended use need to address both individual factors (eg, regarding discarding, safe use and duration, and number of uses), and organizational factors (eg, management of the supply chain, safe reprocessing and storage, staff training, and monitoring and evaluation of practice).

Certain steps can be taken to mitigate risk, such as the extending use of single-use masks or respirators before resorting to reuse and regularly inspecting masks and respirators for integrity, visible damage, and fit. Knowing when equipment must be discarded is crucial, as is appropriate storage and clear labelling of respirators between use to avoid cross use between workers. Organizations should ensure that adequate training in this is provided.

Policy guidance emphasizes the need to assess the contagion risk of an encounter and the need to use recommended protective ensemble for that situation.^7^ Surgical masks and filtering face-piece respirators are only 1 component of PPE, which typically includes gloves, long-sleeved fluid repellent gown, and eye protection.^3^ Safe donning and doffing are critical.^3^ PPE is considered a last line of defense within the hierarchy of infection control measures which also includes administrative and environmental and engineering controls.

### Unanswered questions and future research

Several areas warrant further investigation in relation to reprocessing. In the current context, research is needed to explore the impact of respirator decontamination methods for SARS-CoV-2, taking the heterogeneity of models into account. Research that explores the impact of decontamination on important outcomes (eg, respirator fit, user comfort, and safety (eg, increased contagion risk and/or residual toxicity) and the feasibility of reprocessing methods at scale is scarce.

Despite consideration in some guidance documents for the extended use and reuse of surgical masks in crisis capacity situations, evidence is limited. Further research is needed regarding the effects of extended use of masks and respirators on outcomes such as fit and user comfort to determine the number of uses possible and the optimum length of extended use. Our recent review of respirator performance and standards found that all respirator types carry a burden to the user of discomfort and interference with communication.^25^ Houghton et al^26^ recently identified user comfort as an important factor influencing adherence with infection prevention and control guidelines^26^; comfort becomes particularly important when PPE is worn for extended periods.

### Strengths and limitations

This review was conducted to a high standard in adherence with current Cochrane rapid review guidance. Our interdisciplinary research team is a particular strength; we included frontline healthcare workers, expertise in occupational medicine, infection control, respiratory protective equipment design and performance, emergency nursing, evidence synthesis, and an information specialist. To facilitate timeliness, we limited our guidance search to 4 major health organizations, and we did not search exhaustively for primary studies. Recent research has compared international regulations regarding the reuse and extended use of filtering face-piece respirators^9^ and guidance on respiratory protective equipment more broadly.^27^ However, the former did not address surgical masks, and neither study compared recommendations with current evidence. Our study integrates recommendations and evidence for single-use surgical masks and respirators to enable a clearer understanding of current evidence and research gaps to facilitate evidence-informed decision making in this area.

In conclusion, extended use and reuse of single-use surgical masks and respirators (with or without reprocessing) should only be considered in situations of extremely critical shortage. Where extended use or reuse is being practiced, healthcare organizations should ensure that policies and systems are in place to ensure that these practices are carried out as safely as possible and in line with available guidance. Areas of guidance lacking clarity and consistency warrant further attention and investigation.

## Supplementary material

For supplementary material accompanying this paper visit https://doi.org/10.1017/ice.2020.1243. click here to view supplementary material
