# Supplementary material for: Extended use or reuse of single-use surgical masks and filtering face-piece respirators during the coronavirus disease 2019 (COVID-19) pandemic: A rapid systematic review
Source: Infect Control Hosp Epidemiol. 2020 Oct 8:1–9. doi: 10.1017/ice.2020.1243 (PMC7588721; doi:10.1017/ice.2020.1243)
Supplement: Supplementary file 1 [file S0899823X2001243Xsup001.zip › S0899823X2001243Xsup001.docx]

**Appendix 2: Search Strategy - Database(s): Ovid MEDLINE(R) and Epub Ahead of Print, In-Process & Other Non-Indexed Citations, Daily and Versions(R) 1946 to April 28, 2020**

| **#** | **Searches** | **Results** |
| --- | --- | --- |
| 1 | exp Masks/ | 9295 |
| 2 | exp Respiratory Protective Devices/ | 2002 |
| 3 | (respiratory protective devices or mask* or face mask* or facemask* or respiratory protection or respirator* or FFP3 or FFP or N95 or N 95 or PAPR or air purifying respirator or filtering face piece).tw,kf. | 514098 |
| 4 | (filtering adj3 (facepiece* or face piece*)).tw,kf. | 269 |
| 5 | or/1-4 | 516692 |
| 6 | Equipment Contamination/ | 10874 |
| 7 | exp Infection Control/ | 62917 |
| 8 | (infection control* or decontaminat* or resanitiz* or resanitis* or desaniti* or contaminat* or antisept* or biocid* or steriliz* or sanitize* or bleach* or hypochlor* or ozon* or ultraviolet or uv).tw,kf. | 504524 |
| 9 | Sodium Hypochlorite/ | 4683 |
| 10 | Disinfectants/ | 12900 |
| 11 | Hydrogen Peroxide/ | 57651 |
| 12 | (clorox or antiformin or oxygenated water or hydrogen peroxyde or hydroperoxide or peroxygen or sodium hypochlorite).tw,kf. | 15306 |
| 13 | Ethanol/ | 86881 |
| 14 | 2-Propanol/ | 1613 |
| 15 | Ethylene Oxide/ | 2827 |
| 16 | (ethanol* or isopropanol or iso-propanol or 2-propanol or isopropyl alcohol or ethylene oxid* or oxirane or rubbing alcohol*).tw,kf. | 149448 |
| 17 | or/6-16 | 783856 |
| 18 | (washable or rewash* or reusable or reprocess* or reus* or reusing or repurpose or recycl* or multiple use* or multiple usage or used again or used repeat* or repeat* use* or repeat* usag* or repeatedly use* or extended usage).tw,kf. | 79563 |
| 19 | 5 and 17 and 18 | 207 |
