# Supplementary material for: Extended use or reuse of single-use surgical masks and filtering face-piece respirators during the coronavirus disease 2019 (COVID-19) pandemic: A rapid systematic review
Source: Infect Control Hosp Epidemiol. 2020 Oct 8:1–9. doi: 10.1017/ice.2020.1243 (PMC7588721; doi:10.1017/ice.2020.1243)
Supplement: Supplementary file 1 [file S0899823X2001243Xsup001.zip › S0899823X2001243Xsup003.docx]

Appendix 5 Systematic reviews of studies exploring re-use, extended use or reprocessing of single-use surgical masks or respirators

| **Review characteristics** | | | **Search strategy** | | **Included studies** | | | **Review findings** |
| --- | --- | --- | --- | --- | --- | --- | --- | --- |
| **Reference (Author, Year)** | **Component** | **Aim (s)** | **Search date** | **Sources searched (number)** | **Number of studies included** | **Methods evaluated (number of types)** | **Outcomes evaluated (number of studies)** | **Summary** |
| Gertsman et al. (2020)^14^ | N95 filtering facepiece respirators | To collect and synthesize existing information on decontamination of N95 filtering facepiece respirators using microwave and heat-based treatments | Inception to March 29th 2020 | Databases (n=8), pre-print servers (n=2), journal hand-searching (n=2) | 11 | - Moist microwave irradiation (microwave-generated steam) (n=6) - Dry microwave irradiation (n=2) - Moist heat incubation (n=5) - Steam (autoclave) (n=3) - Dry heat (n=3) | **Decontamination effectiveness**   - Germicidal effects (n=7)   **Respirator Performance and Appearance**   - Filtration efficiency (n=6) - Airflow resistance (n=3) - Physical integrity (n=7)   **Fit, User Comfort and Safety**   - Fit (n=2) - Odour (n=3) | - Moist/dry microwave irradiation and moist/dry heat between 60-90^o^C can effectively sterilize certain N95 filtering facepiece respirator models while maintaining mask function within acceptable ranges - Use of high heat (> 90^o^C) and autoclaving are not supported by included studies as these interventions more consistently compromised mask integrity - Risk of bias across all outcomes relatively low - Few studies investigated fit - No study conducted using SARS-CoV-2 - Heterogeneity in conditions under which microwave irradiation and heat applied - Variability in mask models used with significant variability of results dependent on mask model used |
| O'Hearn et al. (2020a)^16^ | N95 (including SN95) filtering facemask respirators | To synthesize existing data on the effectiveness and safety of using disinfectants to decontaminate N95 filtering facepiece respirators | Inception to March 31st 2020 | Databases (n=8), pre-print servers (n=2), journal hand-searching (n=2) | 13 | - Sodium hypochlorite (n = 21) - Liquid hydrogen peroxide (liquid H2O2, n = 4) - Vaporized hydrogen peroxide (vaporized H2O2, n = 12) - Ethanol (n = 9) - Isopropyl alcohol (n = 3) - Ethylene oxide (EtO, n = 6) - Other (n = 3) | **Decontamination effectiveness**   - Germicidal effects (n=8)   **Respirator Performance and Appearance**   - Filtration efficiency (n=5) - Airflow resistance (n=3) - Physical integrity (n = 6)   **Fit, User Comfort and Safety**   - Fit (n = 2) - Safety/irritation (n=3) - Odour (n=3) | - A single cycle of vaporized hydrogen peroxide removes infectious pathogens without affecting mask function or fit, and with little change in filtering facepiece respirators’ physical appearance - Further research is required before the acceptability of decontamination using liquid hydrogen peroxide can be determined - Sodium hypochlorite, ethanol, isopropyl alcohol and Ethylene oxide are not recommended due to safety concerns and/or adverse effects on mask function (e.g. odour) - Only two studies assessed impact on mask fit - Two studies conducted using SARS-CoV-2 - Overall risk of bias in results for aerosol penetration, airflow resistance, and fit were low in all studies. Moderate overall risk of bias noted for germicidal outcomes - Each study used a different combination of mask types - All evaluations done on new, unworn filtering facepiece respirators. Unclear whether extended use prior to decontamination would alter findings |
| O'Hearn et al. (2020b)^15^ | N95 (including SN95) filtering facemask respirators or their components | To synthesize existing data on the effectiveness of ultraviolet germicidal irradiation on N95 filtering facepiece respirator decontamination. | Inception to March 24th 2020 | Databases (n=5) | 13 | - Ultraviolet germicidal irradiation (UVGI) | **Decontamination effectiveness**   - Germicidal effects (n=7)   **Respirator Performance and Appearance**   - Filtration efficiency (n=5) - Airflow resistance (n=3) - Physical integrity (n=6)   **Fit, User Comfort and Safety**   - Fit (n=2) - Odour (n=3) - Perceived donning ease/comfort (n=1) | - A single cycle of UVGI with UV-C light (ideally 40,000 J/m2) decontaminated mask surfaces exposed to viruses in laboratory conditions without significant changes in filtering facemask respirators appearance or odor and does not affect function (e.g. particle penetration, airflow resistance) - Level of decontamination was associated with cumulative UV dose and conditions used to simulate viral spread, e.g. addition of salts and biological particulate (saliva and protein) - Only two studies evaluated physical characteristics or fit following UVGI exposure; they did not present evidence of negative effects - Number of decontamination and re-use cycles that can be applied to a filtering facepiece respirators will be limited by breakdown imposed by both UVGI and donning and doffing - Cumulative dose of at least 20,000 and ideally 40,000 J/m2 should be used for clinical application of UVGI or further investigation. Data from one study that evaluated mask fit following a comparable dose of UVGI (32,400 J/m2) showed no change in mask fit, but additional investigation needed - No summary statement provided for risk of bias of included studies; however individual judgements provided show studies to have mostly low risk of bias across domains; except for blinding which seemed to be poorly done/unclear - Each study used a different combination of mask types - All evaluations were conducted in laboratory settings and do not represent real world conditions - No study conducted using SARS-CoV-2 |
| Zorko et al. 2020^25^ | Medical/ Surgical facemasks | To evaluate and synthesize the evidence on decontamination or sterilization interventions for the purpose of surgical mask re-use. | Inception to April 8, 2020 | Databases (n=8), pre-print servers (n=2), journal hand-searching (n=2) | 7 | *Decontamination interventions after use to enable re-use (n=1)*   - Dry heat (via rice cooker) - High-pressure moist heat (i.e. autoclave) - 70% ethanol - 100% isopropanol - 0.5% sodium hypochlorite [i.e. bleach]   *Interventions applied before use to enable potential re-use or extended use (n=6)*   - Antimicrobial interventions (n=4) - Fluorochemical repellent (n=1) | *Decontamination interventions after use to enable re-use (n=1)*   - Filtration efficiency - Airflow resistance - Physical characteristics   *Interventions applied before use to enable potential re-use or extended use*   - Germicidal effects (n=4) - Filtration efficiency (n=4) - Airflow resistance (n=3) - Physical characteristics/ adverse effects (n=2) | - Dry heat may alter surgical mask performance less than high-pressure moist heat or chemical interventions; however, the germicidal effect of dry heat in surgical masks is unclear - Bleach is not a safe method of decontaminating surgical masks; surgical mask performance is significantly altered and safety data from N95 filtering facepiece respirators studies suggest potential health risks associated with off-gassing - Inadequate evidence on the safety or efficacy of any decontamination intervention for extended use or re-use of surgical masks in the clinical setting - Included studies used heterogeneous interventions, methodologies and outcome assessments - No studies provided information on feasibility or resource requirements, or applicability within real-life settings - No study done with SARS-CoV-2 |
