# Supplementary material for: Extended use or reuse of single-use surgical masks and filtering face-piece respirators during the coronavirus disease 2019 (COVID-19) pandemic: A rapid systematic review
Source: Infect Control Hosp Epidemiol. 2020 Oct 8:1–9. doi: 10.1017/ice.2020.1243 (PMC7588721; doi:10.1017/ice.2020.1243)
Supplement: Supplementary file 1 [file S0899823X2001243Xsup001.zip › S0899823X2001243Xsup005.docx]

**Appendix 3: Included guidance documents**

| **Organisation or Institution / Country or jurisdiction** | **Title / Date of publication** | **Scope** | **Components addressed** | **Definition of re-use** | **Definition of extended use** |
| --- | --- | --- | --- | --- | --- |
| CDC (USA) | Strategies for Optimizing the Supply of Facemasks^10^  17-Mar-20 | Strategies or options to optimize supplies of facemasks in healthcare settings when there is limited supply. | Surgical masks | Re-use: ‘The practice of using the same respirator/mask by one healthcare worker for multiple encounters with patients but removing it (‘doffing’) after each encounter. The respirator is stored in between encounters to be put on again (‘donned’) prior to the next encounter with a patient.’ | ‘The practice of wearing the same respirator/mask for repeated close contact encounters with several patients, without removing the respirator/ mask between patient encounters’ |
| CDC (USA) | Recommended Guidance for Extended Use and Limited Re-use of N95 Filtering Facepiece Respirators in Healthcare Settings^4^  27-Mar-20 | Recommends practices for extended use and limited re-use of NIOSH-certified N95 filtering facepiece respirators. Intended for use by professionals who manage respiratory protection programs in healthcare institutions. | Filtering Facepiece Respirators (N95/FFP2 only) |  |  |
| CDC (USA) | Coronavirus Disease 2019: Decontamination and Re-use of Filtering Facepiece Respirators^11^  09-Apr-20 (with minor updates on 29-Apr-2020) | Summarizes research about decontamination of FFRs before re-use. | Filtering facepiece respirators (i.e. N95/FFP2 or FFP3 standard or equivalent) |  |  |
| ECDC (Europe) | Cloth masks and mask sterilisation as options in case of shortage of surgical masks and respirators^12^  26-Mar-20 | Use of cloth face masks and sterilisation of respirators and surgical masks in healthcare settings with COVID-19 cases if shortage of surgical masks and respirators. | Single-use respirators (FFP2 and FFP3) | Not defined | Not defined |
| PHE (UK) | Considerations for acute personal protective equipment (PPE) shortages^13^  27-Apr-20 (with minor updates on 21-May-20) | Sessional use and re-use of PPE when there are severe shortages of supply. | Fluid-repellent surgical face masks and disposable respirators (FFP3 & FFP2/N95) | Re-use: ‘Using the same item again, with appropriate precautions, by the same healthcare worker’ | **‘**Sessional use by one health or care worker during one shift while working’ |
| WHO (World) | Rational use of personal protective equipment for coronavirus disease (COVID-19) and considerations during severe shortages^5^  06-Apr-20 | Rational use of PPE in health care and home care settings, and during handling of cargo; current disruption of global supply chain and considerations for decision making during severe shortages of PPE. | Surgical masks & filtering facepiece respirators (N95/FFP2 or FFP3 standard or equivalent) | Re-use not defined.  Reprocessing: ‘decontamination using disinfection or sterilization methods followed by re-use of either reusable or disposable PPE’ | ‘Using for longer periods of time than normal without removing (for up to 6h) when caring for a cohort of COVID-19 patients’ |

CDC: Centers for Disease Control and Prevention (US), ECDC: European Centre for Disease Prevention and Control, FFP: Filtering Facepiece, PHE: Public Health England, PPE: Personal protective equipment, WHO: World Health Organization
